# Limited Air Pollution Research on the African Continent: Time to Fill the Gap

**DOI:** 10.3390/ijerph19116359

**Published:** 2022-05-24

**Authors:** Christina H. Fuller, A. Kofi Amegah

**Affiliations:** 1 Department of Population Health Sciences, Georgia State University School of Public Health, Atlanta, GA 30303, USA; 2 Public Health Research Group, Department of Biomedical Sciences, University of Cape Coast, Cape Coast, Ghana

Air pollution is a major threat to human health and well-being, and improving air quality is necessary to achieve the sustainable development goals [1,2]. Air quality in African cities has deteriorated and can be partially attributed to rapid population growth and industrial expansion in cities [1,3]. Some of the highest fine particle levels in the world have been recorded in cities of sub-Saharan Africa (SSA) and other developing regions [4]. Fine particle (PM_2.5_) concentrations in SSA cities has been estimated at around 100 µg/m^3^ compared to <20 µg/m^3^ in most European and North American cities [4]. Air pollution is the fifth leading risk factor for mortality worldwide [5] and is responsible for over 700,000 deaths annually in Africa [6]. Additionally, according to 2010 estimates, 626,000 DALYs in Africa can be attributed to ambient PM_2.5_ exposure [7]. Amegah and Agyei-Mensah (2017) indicated that the estimates for Africa are likely to be higher than reported owing to the limited air pollution epidemiological data emanating from the region. This perception is affirmed by the fact that estimates of the burden of disease attributable to air pollution exposure in Africa are based on integrated exposure–response (IER) model that are derived largely from studies conducted in Europe and North America, locations with low ambient PM_2.5_ levels compared to what pertains in Africa [8]. According to Akumu [9], the cost of air pollution in African cities could be as high as 2.7% of gross domestic product.

Addressing the worsening air pollution problem in Africa is proving increasingly difficult due in part to the lack of data on air pollution concentrations in countries and the magnitude of the associated health impacts [3]. Air quality monitoring networks are weak and limited on the continent, making assessment of exposure and health burden challenging. Often priority is given to other concerns such as water supply, sanitation, malaria and infectious disease control for which there is substantial evidence of its public health relevance. Therefore, it is extremely difficult to convince governments to invest in air quality monitoring and other pollution control measures. This worrying situation calls for investments in air quality monitoring by governments to bridge the huge air quality data gap; building capacity of health workers to mount surveillance for air pollution related diseases; resourcing environmental protection agencies to develop air quality standards and ensure compliance; and encouraging international funding organizations to institute funding mechanisms targeted to African researchers.

This issue of *IJERPH* is designed to highlight research on air pollution conducted in African countries. The intent is to inform the scientific community on the extent of the air pollution problem and the associated health burden in these populations that has previously been overlooked. We seek to galvanize new efforts to build air pollution monitoring and research capacity, showcase air pollution data, and conduct health impact assessment within African countries. The majority of articles in this issue are written by African researchers, which is necessary for authentic representation as well as acknowledgment of the existing capacity to conduct robust research. Accepted papers in this Special Issue include air pollution monitoring and modelling, exposure assessment, and epidemiology from the countries of Kenya, Ethiopia and South Africa and the continent as a whole.

The Kenyan-based study assessed long-term PM_2.5_ exposure among children under five years of age and investigated their association with symptoms of acute respiratory infections. The study conducted in Ethiopia investigated the levels of PM_2.5_ from burning solid fuels in rural households of Butajira. Of the two studies conducted in South Africa, one examined the spatial and temporal variations in PM_10_ concentrations between 2010–2017 from monitoring 44 sites across four provinces of the country namely (Gauteng, Mpumalanga, Western Cape and KwaZulu-Natal). The other study investigated ambient air pollution exposure among adults residing in four informal settlements of the Western Province of South Africa and association with cardiorespiratory outcomes. The final study utilized a continent-wide combined grid and administrative units to explore the spatio-temporal change, spatial dependence patterns, and evolution trend of PM_2.5_ concentrations and multi-dimensional urbanization in Africa for the period 2000–2018.

This Special Issue serves a purpose of elevating the conversation around air pollution measurement and health impacts on the African continent. In addition, the selection of manuscripts highlights the scholarship of many Africa-based researchers. We intend for this issue to catalyze further research to expand monitoring networks and epidemiological studies across the nations of the African continent.
